# Comparison of the surgical treatment strategies for Siewert type II squamous cell carcinoma in the same area as esophagogastric junction carcinoma: data from a single Japanese high-volume cancer center

**DOI:** 10.1007/s00595-013-0773-4

**Published:** 2013-10-29

**Authors:** Hiroshi Yabusaki, Atsushi Nashimoto, Atsushi Matsuki, Masaki Aizawa

**Affiliations:** Department of Digestive Surgery, Niigata Cancer Center Hospital, 2-15-3 Kawagishicyo, Chuo-ku, Niigata, 951-8566 Japan

**Keywords:** Siewert type II, Squamous cell carcinoma, Surgical treatment

## Abstract

**Purpose:**

Siewert type II esophagogastric junction adenocarcinoma (ADC) and squamous cell carcinoma (SCC) existing in the same area have distinct clinicopathological characteristics. The objective of this study was to examine differences in the surgical treatment and survival data, according to the histological subtype, in a single high-volume cancer center.

**Methods:**

We retrospectively examined data from a total of 123 patients. Seventy-two patients with Siewert type II ADC and 51 patients with SCC in the same area.

**Results:**

In terms of the clinicopathological factors, the SCC patients had more advanced stage disease and thoracotomy was more frequently performed than in the ADC patients. The 5-year overall survival (OS) rates did not differ significantly between SCC and ADC, regardless of whether or not mediastinal, splenic hilum and para-aortic lymph node dissection was performed. Based on the calculated index for the frequency of nodal metastasis and the five-year OS rate for involvement at each level, only node nos. 1, 2, 3 and 7 had a high index (>5) in both groups. The multivariate Cox regression analysis showed that only age (<65), the pN category and residual tumor classification were independently associated with the outcome.

**Conclusions:**

Differences in the histological type of esophagogastric junction cancer were not independent prognostic factors for survival, and there appears to be a benefit to dissecting the number 1, 2, 3 and 7 lymph nodes.

## Introduction

In recent years in Western countries, the dominant histological subtype of carcinoma found in the lower esophagus and esophagogastric junction (EGJ) has shifted from squamous cell carcinoma (SCC) to adenocarcinoma (ADC) [1, 2]. While SCC still accounts for the majority of these malignancies in Japan, the current availability of *Helicobacter pylori* eradication therapy is anticipated to change the proportions of these cancers, giving rise to a trend similar to that observed in Western countries [3].

At the 2nd International Gastric Cancer Congress held in Munich in 1997, a consensus was reached to classify ADC in the EGJ into three subtypes according to the Siewert classification [4]. Using the anatomical classification of the esophagus, ADC of the EGJ was defined as ADC with esophageal invasion with the epicenter of a tumor within 5 cm of the EGJ in the TNM Classification of Malignant Tumors 7th Edition [5] In Japan, Nishi’s classification system is also used to classify carcinoma of the gastric cardia, and cancer at the EGJ is defined as a tumor with the epicenter within 2 cm proximal and distal to the EGJ, regardless of its histological subtype [6–8].

As described above, EGJ carcinoma comprises two histological subtypes, ADC and SCC. ADC and SCC have distinct predisposing risk factors and clinicopathological features. However, the carcinoma subtypes were not distinguished in some of the previous clinical trials, and it is unclear whether the optimal treatments differ among these subtypes [9]. For example, the most appropriate surgical procedures and extents of lymph node dissection for ADC and SCC [10], considered separately in the ESMO Clinical Practice Guidelines for the diagnosis, treatment and follow-up, as well as in the NCCN Clinical Practice Guidelines in Oncology, have not yet been established. It is of the utmost importance to investigate the biological characteristics of ADC and SCC, and to identify the optimal treatment strategies for these distinct EGJ carcinomas [11].

Type II tumors, carcinomas of the true cardia, with the epicenter within an area 1 cm above and 2 cm below the cardia, in particular, are most likely to contain both ADC and SCC. Histologically specific treatment strategies, like those used in lung cancer and urinary bladder carcinoma, may be an important clinical issue, especially for SCC occurring at the same site as ADC. The objectives of this study were to examine the differences between SCC and ADC in terms of the surgical treatment, lymph node metastasis status and survival data, based on the histological subtype, in a single Japanese high-volume cancer center.

## Methods

We diagnosed type II EGJ carcinoma if the epicenter was within 1 cm proximal and 2 cm distal to the anatomical EGJ based on a photograph of the resected specimen [12]. Between January 1985 and December 2008, a total of 6356 patients, 5658 patients with gastric carcinoma and 698 with esophageal carcinoma, underwent surgery at the Division of Surgery, Niigata Cancer Center Hospital, Niigata, Japan. We retrospectively examined the data from a total of 123 of these patients (72 with Siewert type II carcinoma undergoing at least D1 lymph node dissection and 51 patients with SCC in the same area with the lesion extending to the esophagus and stomach).

The tumor staging and nodal classification were performed according to the International Union Against Cancer (UICC) TNM staging system for EGJ cancer [5]. The lymph node levels were numbered according to the definition established by the Japanese Gastric Cancer Association and Japanese Esophageal Society [7, 8].

### Surgical procedures

In principle, proximal or total gastrectomy without splenectomy via the abdominal approach was carried out for cT1 carcinoma, and thoracic esophagectomy or total gastrectomy with or without splenectomy via the thoracic or abdominal approach was carried out for cT2–T4 carcinoma. All procedural decisions were made by the primary surgeon.

### Statistical analysis

Variables were expressed as the mean ± SD. Comparisons between groups were performed with Student’s *t* test, the χ^2^ test and the Mann–Whitney *U* nonparametric test. The multivariate analyses using Cox’s proportional hazards model were performed to identify independent prognostic factors. The calculated mean survival time (MST) and the 5-year overall survival (OS) rates were calculated from the initiation of surgery until death. A survival analysis was performed using the Kaplan–Meier method. The log-rank test was used to calculate the statistical significance of the differences in OS rates between groups. A two-tailed value of *p* < 0.05 was considered to indicate a statistically significant difference. We evaluated the therapeutic benefit obtained by node dissection at each lymph node level, based on the index of the estimated benefit of lymph node dissection calculated by multiplying the incidence of metastasis by the 5-year OS rate of patients with metastasis at each node level [13].

## Results

### Patient backgrounds and surgical procedures

With regard to the clinicopathological factors, SCC had more invasive characteristics, including more extensive esophageal invasion, deeper tumor invasion and more advanced pathological stages, than ADC. Furthermore, the intestinal type was more frequently observed in SCC patients (Table 1).

**Table 1:** Demographics and surgical procedures of the 123 patients with EGJ carcinoma

	SCC (51)	ADC (72)	*p* value
Tumor size (cm)	5.8 ± 2.0	5.3 ± 2.7	0.2839
Length of esophageal invasion (cm)	3.1 ± 1.8	1.8 ± 1.2	<0.0001
Macroscopic type			
Borrmann Type 1, 2	47 (92.2)	42 (58.3)	
Borrmann Type 3, 4	4 (7.8)	30 (41.7)	<0.0001
Histological type			
Differentiated type	37 (72.5)	48 (66.7)	
Undifferentiated type	14 (27.5)	24 (33.3)	0.5392
Depth of tumor invasion			
pT1/2	6 (11.8)	37 (51.4)	
pT3/4	45 (88.2)	35 (48.6)	<0.0001
Lymph node metastasis			
Negative	17 (33.3)	27 (37.5)	
Positive	34 (66.7)	45 (62.5)	0.5546
Peritoneal metastasis			
Negative	51 (100)	70 (97.2)	
Positive	0 (0.0)	2 (2.8)	0.2462
Liver metastasis			
Negative	51 (100)	70 (97.2)	
Positive	0 (0.0)	2 (52.8)	0.2462
Venous invasion			
Negative	25 (49.0)	29 (40.3)	
Positive	26 (51.0)	43 (59.7)	0.2550
Lymphatic invasion			
Negative	10 (19.6)	17 (23.6)	
Positive	41 (80.4)	55 (76.4)	0.6089
Stage			
I/II	18 (35.3)	37 (51.4)	
III/IV	33 (64.7)	35 (48.6)	0.0004
Residual tumor			
R0	46 (90.2)	67 (93.1)	
R1/2	5 (9.8)	5 (6.9)	0.6133
Length of operation (min)	249 ± 63	225 ± 88	0.3470
Blood loss (ml)	216 ± 150	259 ± 217	0.5095
Approaches			
Right thoracotomy	11 (21.6)	2 (2.8)	<0.0001
Left thoracophrenicolaparotomy	25 (49.0)	20 (27.8)	0.0035
Laparotomy	15 (29.4)	50 (69.4)	<0.0001
Combined resection			
Spleen	23 (43.1)	42 (58.3)	0.0474
Pancreas	5 (9.8)	21 (29.2)	0.0011

(); %

*pT* pathological depth of tumor invasion, *pT1* invasion of the mucosa or submucosa, *pT2* invasion of the muscularis propria, *pT3* invasion of the subserosa, *pT4* invasion of the serosa

Thoracic esophagectomy via right thoracotomy or a left thoracoabdominal (TA) approach was more frequently performed in SCC patients, whereas total gastrectomy with caudal pancreatectomy and splenectomy via the abdominal-transhiatal (TH) approach were the most common procedures for ADC (Table 1).

### Treatment results and survival

**Fig. 1:**
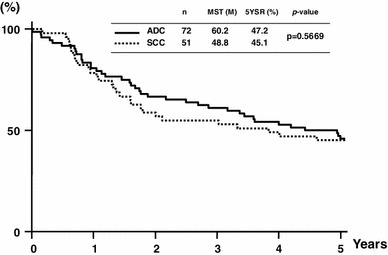
Overall survival after resection of esophagogastric junction carcinoma according to histologic subtype

The median follow-up was 9.0 years (range 3.8–24.8). The MST was 48.8 months, and the 5-year OS rate was 45.1 % for the SCC patients. The corresponding values for the ADC patients were 60.2 months and 47.2 %. Thus, there were no significant survival differences between the SCC and ADC patients (Fig. 1).

The 5-year OS rates also did not differ significantly between SCC and ADC patients with/without dissection of the lower mediastinal lymph nodes, such as Nos. 108, 110, 111 and 112. There were no differences in the five-year OS rates between SCC and ADC patients with/without splenic hilum (No. 10) and para-aortic (No. 16) lymph node dissection.

### Distributions of the metastatic nodes and the index of estimated benefit from lymph node dissection

As shown in Table 2, nodal metastases frequently involved the abdominal lymph nodes, followed in frequency by node Nos. 1, 3, 2 and 7 in both ADC and SCC patients. Mediastinal lymph node dissection was performed in a total of 84 patients, and the metastatic rate was 22.9 % in SCC patients and 13.9 % in ADC patients. The metastatic rate of the No. 10 lymph node was low, at 0 % in SCC and 7.0 % in ADC patients. Only 31 patients underwent No. 16 lymph node dissection, and the metastatic rate was 28.6 % in SCC and 20.8 % in ADC cases (Table 2). Extended lymph node dissection was performed for regions where metastasis was suspected based on the preoperative clinical imaging findings.

**Table 2:** Distribution of the metastatic nodes and index of the estimated benefit from lymph node dissection

Lymph node station	SCC (51)					ADC (72)				
	Dissected cases	Metastasis cases	Metastatic rate	5YSR of metastasis cases	Index	Dissected cases	Metastasis cases	Metastatic rate	5YSR of metastasis cases	Index
1	49	20	40.8	27.8	11.3	72	33	45.8	24.2	11.1
2	49	9	18.4	33.3	6.1	72	16	22.2	37.5	8.3
3	49	17	34.7	33.3	11.6	72	29	40.3	27.6	11.1
4s	37	0	0	–	0.0	57	1	1.8	0	0.0
4d	35	0	0	–	0.0	66	2	3.0	50	1.5
5	31	0	0	–	0.0	60	1	1.7	0	0.0
6	36	1	2.8	0	0.0	63	3	4.8	33.3	1.6
7	47	9	19.1	33.3	6.4	71	20	28.2	20	5.6
8a	44	1	2.3	0	0.0	66	5	7.6	20	1.5
9	45	4	8.9	0	0.0	69	11	15.9	9.1	1.4
10	24	0	0	–	0.0	43	3	7.0	33.3	2.3
11p	39	5	12.8	20.0	2.6	64	6	9.4	16.7	1.6
11d	30	0	0	–	0.0	40	0	0	–	0.0
12a	6	1	16.7	0	0.0	36	0	0	–	0.0
16	7	2	28.6	0	0.0	24	5	20.8	20	4.2
a2lat	6	1	16.7	0	0.0	21	5	23.8	20	4.8
a2int	1	1	100	–	0.0	3	1	33.3	0	0.0
b1lat	4	1	25.0	0	0.0	10	1	10.0	0	0.0
b1int	0	0	0	–	0.0	6	0	0	–	0.0
ML	48	15	31.3	9.1	2.1	36	5	13.9	20	2.8
108	21	1	4.8	50.0	2.4	9	0	0	–	0.0
110	46	14	30.4	14.7	4.5	34	4	11.7	25	2.9
111	36	3	8.3	0	0.0	32	0	0	–	0.0
112	12	1	8.3	0	0.0	8	1	12.5	0	0.0

An index of the benefit gained by the dissection of each station was calculated by multiplication of the frequency of metastasis at the station by the 5-year survival rate of patients with metastasis at that station; metastatic rate × 5-year OS/100

Based on the index calculated employing the frequency of nodal metastasis and the 5-year OS rate for involvement at each lymph node level, only node Nos. 1, 2, 3 and 7, in both SCC and ADC patients, had a high index (>5). Although the estimated therapeutic index of lymph node dissection was 5 or less, the dissection of No. 110 in SCC and dissection of No. 16a2 lat in ADC patients were found to be effective (Table 2).

### Lymph node metastasis status, recurrence sites and the results of the multivariate cox regression analysis

In 16 patients with mediastinal lymph node metastasis, the average total number of metastatic lymph nodes was 6.7, which was significantly higher than that (2.5) in the 68 patients who were positive for metastasis to only the abdominal lymph nodes. Similar results were obtained when metastases were examined according to the histological subtypes of SCC and ADC (Table 3).

**Table 3:** The total number of cases with lymph node metastasis with and without mediastinal lymph node metastasis

	All *n* = 84	SCC *n* = 48	ADC *n* = 36
Mediastinal LN metastasis (+)	6.7 ± 5.8	5.3 ± 3.5	9.8 ± 8.8
Mediastinal LN metastasis (−)	2.5 ± 3.6	2.0 ± 3.1	3.0 ± 4.1
*p* value	0.0003	0.0047	0.0063

Hematogenous metastasis was noted in 25 (10 SCC and 15 ADC) patients, and liver metastasis accounted for 17 of these patients. Lymphatic metastasis was observed in 14 (11 SCC and 3 ADC) patients; No. 16 lymph node metastasis in six patients, and mediastinal and cervical lymph node metastases in three patients each (Table 4).

**Table 4:** The number of patients with each site of first recurrence

	SCC	ADC	Total
Hematogenous	10	15	25
Liver	8	9	17
Lung	0	3	3
Bone	2	1	3
Brain	0	1	1
Skin	0	1	1
Lymphatic	11	3	14
Para-aortic	5	1	6
Mediastinal	3	0	3
Cervical	2	1	3
Other abdominal	1	1	2
Peritoneal	1	8	9
Local	1	0	1

A multivariate Cox regression analysis showed that only the age (<65 years), pN category (pN0) and residual tumor classification (R0) were independently associated with the outcome. Neither the histological subtype nor lower mediastinal, No. 10 and 16 node dissections were independently associated with the outcomes (Table 5).

**Table 5:** The results of the multivariate Cox regression analysis for the overall survival in patients with EGJ carcinoma (*n* = 123)

Variables	Hazard ratio	95 % confidence limits	*p* value
Age (< 65/≥ 65)	0.365	(0.215–0.618)	<0.01
Lymph node metastasis (n (−)/n (+))	0.370	(0.205–0.666)	<0.01
D-number (D0/D1, D2)	0.398	(0.158–0.998)	<0.01

## Discussion

No standard procedure has yet been established for the surgical treatment of EGJ carcinoma in terms of the presence/absence of the need for thoracotomy, extent of esophageal and gastric resection, extent of mediastinal and abdominal lymph node dissection and the need for splenectomy. In the present study, we identified clear differences in the clinicopathological factors, approaches and surgical procedures used for SCC and ADC in our center.

A Dutch trial involving patients with Siewert type I/II carcinoma, treated in two high-volume centers, examined the superiority of two-field lymphadenectomy via the right TA over D1 lymphadenectomy via the TH approach [14]. It was recommended that right TA be performed for patients with type I tumors and TH for those with type II carcinoma based on a subsequent subset analysis [15].

In Japan, a randomized controlled trial (RCT) was conducted by the Stomach Cancer Study Group of the Japan Clinical Oncology Group to compare the left TA approach with the abdominal-TH approach in patients with Siewert Type II/III carcinoma (JCOG9502) [16]. The results failed to demonstrate the superiority of the left TA approach in terms of the OS. Accordingly, it was concluded that the abdominal-TH approach with para-esophageal lymph node dissection to a feasible extent should be recommended for Siewert Type II/III tumors.

Moreover, based on a study involving 1,002 patients, Siewert et al. [17] justified applying right TA for type I carcinoma of the esophagus and the abdominal-TH approach and D2 dissection of abdominal lymph nodes for type II and III gastric tumors. In addition, Yamashita et al. [18] examined the optimal extent of lymph node dissection for Siewert type II carcinoma in a study including 225 patients, and determined that dissection of the paracardial and lesser curvature nodes is essential for achieving the therapeutic benefit of surgery. However, all of these studies were conducted for ADC. Therefore, further studies are needed to investigate the effects of histological differences on the distribution of lymph node metastasis and outcomes. However, to the best of our knowledge, there have been no reports on the surgical procedures or survival data based on the tumor histology of EGJ carcinoma.

The survival data in our series included a MST of 60.2 months and a 5-year OS rate of 47.2 % for ADC patients. The index calculated employing the frequency of nodal metastasis and the 5-year OS rate for involvement at each lymph node level indicated that the only lymph nodes which should be dissected were Nos. 1, 2, 3 and 7 in ADC patients. The multivariate Cox regression analysis showed that age, the pN category and the residual tumor classification were independently associated with the outcome. These results are in good agreement with those obtained in other studies [14, 16–19]. Therefore, the data from our series are highly consistent with those of previous studies, indicating the reliability of our present investigation.

In our series, the clinicopathological background factors and surgical procedures differed between the SCC and ADC groups, while there were no significant differences in the outcomes or therapeutic benefits provided by lymph node dissection. However, only three of the 51 SCC patients did not undergo mediastinal lymph node dissection. Because of this possible bias in the data, we cannot directly assess the clinical significance of mediastinal lymph node dissection in SCC cases.

The rate of mediastinal lymph node metastasis in our series was 22.9 % (11/48) in SCC and 13.9 % (5/36) in ADC patients, which was not significantly different. In addition, the values of the index of estimated benefit from the mediastinal lymph node dissection were similar in SCC and AC cases (2.9–2.2).

In our series of 123 patients, none exhibited mediastinal lymph nodes metastasis alone, suggesting that metastasis to mediastinal lymph nodes basically occurs after that to abdominal lymph nodes. In 16 patients with mediastinal lymph nodes metastasis, the average total number of metastatic lymph nodes was 6.7, which was significantly higher than that (2.5) in the 68 patients who were positive only for metastasis to abdominal lymph nodes. Similar results were obtained when the metastases were examined according to the histological subtypes. These results indicate that metastasis of EGJ carcinoma of Siewert type II occurs first to the abdominal lymph nodes, and then to mediastinal lymph nodes, regardless of the histopathological subtype of the tumor. Thus, patients with mediastinal lymph node metastasis probably already have abdominal lymph node metastasis, and the total number of metastatic lymph nodes would inevitably be high. Consequently, the addition of mediastinal lymph node dissection with additional thoracotomy may not provide a meaningful clinical benefit.

Our examination of the recurrence sites revealed that hematogenous recurrence, mainly in the liver, accounted for the majority of relapses in both SCC and AC, followed by No. 16 lymph node recurrence. Only three SCC patients and none of the ADC patients had mediastinal lymph nodes recurrence. This revealed hematogenous metastasis to the liver to be common in EGJ carcinoma cases, an observation consistent with other studies [18, 20].

Perioperative chemo-radiotherapy for EGJ carcinoma, including SCC, reportedly improves the outcomes [9]. Since patients with EGJ carcinoma are potentially at high risk of hematogenous micrometastasis, prophylactic dissection of mediastinal lymph nodes would offer no apparent benefits in terms of the local control or prognostic improvement. Among our patients with mediastinal lymph nodes metastasis, one SCC patient with three metastatic nodes (one at No. 108 and two at No. 110), and only one ADC patient with one metastatic lymph node, at No. 110, survived longer than 5 years. Based on these findings, we speculated that the effectiveness of mediastinal lymph node dissection is nearly as low in SCC as it is in ADC.

## Conclusions

Overall, taking the surgical invasiveness into account, it can be assumed that the appropriate procedures for both SCC and ADC include dissection of the abdominal lymph nodes, focusing on the paracardial area and the lesser curvature of the stomach, para-esophageal lymph nodes (No. 110) for SCC, and a part of the para-aortic lymph nodes (No. 16 a2 lat) for ADE via the abdominal-TH approach.

A multivariate Cox regression analysis showed that histological subtype (SCC and ADE) was not an independent prognostic factor.

In this study, two datasets for esophageal and gastric tumors treated in our center were integrated for the analysis. Thus far, patients with lesions on the esophageal side have undergone esophageal surgery performed by specialists, while those with lesions on the gastric side have been treated by surgeons specializing in gastric surgery. This historical background may have yielded apparently contradictory outcomes. Further evidence is needed to confirm the present findings and establish the outcomes of each of the skilled approaches used for SCC and ADC. Such evidence is needed to prepare for the anticipated increase in the number of patients with EGJ carcinoma.
